# The determinants of leptin, angiopoietin like 8, and thyroid hormones levels in Saudi females with type 2 diabetes mellitus: A retrospective study

**DOI:** 10.1097/MD.0000000000039339

**Published:** 2024-09-06

**Authors:** Dalal Binjawhar, Walaa Mohammedsaeed

**Affiliations:** a Department of Chemistry, College of Science, Princess Nourah Bint Abdulrahman University, Riyadh, Saudi Arabia; b Department of Clinical Laboratory Science, Faculty of Applied Medical Science at Taibah University, Al Madinah, Saudi Arabia.

**Keywords:** ANGPTL8, hyperthyroidism, hypothyroidism, insulin resistance, leptin, obesity, type 2 diabetes mellitus

## Abstract

This study aimed to assess the prevalence of thyroid dysfunction, as measured by hormone levels, in Saudi women with type 2 diabetes mellitus (T2DM). The study will also assess thyroid hormones and leptin, angiopoietin like 8 (ANGPTL8), obesity, and cardiovascular diseases (CVD) in T2D patients. A total of 250 women aged 40 to 60 years with T2DM were retrospectively studied between 2021 and 2022. This research examined medical records for T2DM patients. In this investigation, no T2DM patients had thyroid autoantibodies in their medical records. These patients were chosen for their FT4 and TSH values. All participants were Saudi females with T2DM, aged 54.5 years. Of the 250 participants, 32% had hypothyroidism, 14.8% had hyperthyroidism, and 40.8% (102) had no thyroid disease. Hypothyroidism (7.8 ± 0.67 mmol/L) exhibited greater fasting blood glucose (FBG) levels than hyperthyroidism (7.1 ± 0.64 mmol/L) (*P* < .05). Hypothyroid and hyperthyroid females had significant differences in high density lipoprotein-cholestrol (HDL-C), triglycerides, triglyceride glucose (TyG) index, body mass index (BMI), waist circumstance (WC), high-sensitivity C-reactive protein (hs-CRP), leptin, ANGPTL8, insulin resistance (IR), and insulin levels (*P* < .05). Pearson’s correlation test showed that T2DM patients’ HDL-C levels were favorably but negatively correlated with leptin and ANGPTL8 levels. In hypothyroidism, thyroid stimulation hormone (TSH) is favorably linked with glycated hemoglobin (HbA1c), triglyscride (TG), TyG index, BMI, WC, leptin, ANGPTL8, hs-CRP, and IR. T2DM is linked to thyroid malfunction, notably hypothyroidism, which correlates positively with TSH. TSH variations due to increasing leptin, ANGPTL8, and TyG index may enhance the risk of insulin resistance diseases, such as obesity and CVD, in Saudi females with T2DM.

## 1. Introduction

Type 2 diabetes mellitus (T2DM) is one of the most prevalent chronic diseases globally, and its incidence is gradually increasing, mainly in developing countries. For example, the incidence of T2DM has increased from 8.5% in 1992 to 39.5% in 2022 in the Saudi population.^[1]^ The pathological characteristics of T2DM are related to increased intestinal glucose absorption, reduced insulin secretion, and enhanced IR, which are considered essential components of hyperthyroidism and hypothyroidism.^[2,3]^

Thyroid hormones has been demonstrated to influence glycogen and gluconeogenesis as well as pancreatic function.^[4]^ The 2 key thyroid hormones are thyroxine (T4) and triiodothyronine (T3), which are controlled by a feedback loop system that releases thyroid-releasing hormone (TRH) in sequence to accelerate the pituitary gland to produce and release thyroid stimulation hormone (TSH). TSH then prompts the thyroid gland to produce T4 and T3.^[4]^

It has been reported that patients with T2DM have an increased incidence of thyroid dysfunction (TD), which is often detected by abnormal thyroid function test results, compared with those without diabetes.^[5]^ Thyroid hormones directly affect insulin secretion. Hypothyroidism, a common TD among patients with diabetes, may reduce insulin production. Hyperthyroidism increases beta-cell glucose sensitivity due to increased beta-cell mass and insulin clearance.^[6]^ Hypothyroidism and hyperthyroidism both affect insulin metabolism and cause IR.^[7]^ Diabetes may impede thyroid function by affecting TSH levels in the hypothalamus and T4 to T3 conversion in peripheral tissues.^[5]^ Studies have linked T2DM to TD.^[8]^ Research at King Abdul-Aziz University Hospital (KAUH) identified a significant link between hypothyroidism and T2DM, noting that T2DM was more prevalent among patients with hypothyroidism (77.2%) than in controls (22.8%).^[9]^ Another study reported that 30.7% of T2DM patients had hypothyroidism in Jeddah, Saudi Arabia.^[10]^ In Saudi Arabia, TD is increasing, especially among women.^[10,11]^ There is inadequate information regarding TD in Saudi females, which is linked to T2DM. As mentioned above, IR is a condition that manifests in both hypothyroidism and hyperthyroidism. Large population-based studies have shown that alterations in T3, T4, and TSH levels can be risk biomarkers linked to a series of cardiometabolic alterations involving central obesity, elevated Blood pressure (BP), hyperuricemia, IR, inflammation, and dyslipidemia.^[12,13]^

Furthermore, an association between obesity and T2DM has been identified for decades, and the most important basis for this relationship is the ability of obesity to stimulate IR. Multiple studies have shown that being overweight or obese is one of the greatest risks for developing T2DM. By 2022, it’s projected that 41% of men and 78% of females in Saudi Arabia’s adult population would be overweight.^[14,15]^ In addition, it is well-known that obesity is correlated with fluctuations in TSH and thyroid hormones; and is also associated with several endocrine and metabolic diseases.^[16]^ Some studies have suggested that leptin (a metabolic regulatory hormone) appears to be a link between obesity and variations in thyroid hormones because leptin concentrations affect TSH release.^[17]^ Researchers have studied the association between leptin levels, obesity, cardiovascular disease (CVD), and T2DM, demonstrating that augmented leptin levels are associated with high IR, high atherogenic plasma index, which is an essential index of CVD, and high body mass index (BMI), which is an obesity indicator among female with T2DM in Madinah, Saudi Arabia.^[18]^ Another hormone is described as a metabolic regulator that affects energy homeostasis and is known as angiopoietin like 8 (ANGPTL8) hormones. Previous research has demonstrated that the levels of circulating ANGPTL8 were changed in patients with thyroid diseases.^[19]^ For example, a study done by Yang et al reported that circulating ANGPTL8 was elevated in patients with hypothyroidism and subclinical hypothyroidism.^[20]^ Nevertheless, little is known up to this date is being identified about the relationship between the level of ANGPTL8 and hyperthyroidism. Moreover, it was revealed that women with T2DM had a high level of ANGPTL8 which was significantly associated with increases in IR and elevated levels of C-reactive protein (hs-CRP), triglycerides (TG), and BMI.^[21]^ Based on the above data, it was noticed that there is a relationship between thyroid dysfunction, T2DM or its complications, and different biomarkers; however, the topic is still complicated, and published data are controversial. Therefore, the present study aimed to evaluate the prevalence of thyroid dysfunction in Saudi females with T2DM and assess the association between thyroid hormones, leptin, ANGPTL8and T2DM complications, such as obesity and CVD.

## 2. Methods

### 2.1. Study design and population

This retrospective study was conducted in 2021 and 2022, involving a sample of 250 females aged 40 to 60 years who were diagnosed with T2DM based on the criteria established by the World Health Organization (WHO).^[22]^ This study focused on analyzing medical records. None of the patients with T2DM included in the present study had evidence of thyroid autoantibodies in their medical records. These patients were selected based on their thyroid hormone (FT4) and TSH levels. The exclusion criteria for patients included a range of factors, such as a medical history of pituitary illness or thyroid surgery, notable impairments in liver and kidney function, pregnancy, and the use of amiodarone, biotin supplements, beta-blockers, thyroid treatments, and corticosteroids. This study is a secondary analysis of data from a previously approved study.^[18]^ The approval number for this study (IRB 022-22) is the same as the original study to ensure consistency and compliance with the ethical guidelines and protocols established in the initial approval.

### 2.2. Blood samples tests

Three milliliters of blood samples were collected from females who fasted for a minimum of 8 hours overnight in order to test various parameters in Madinah Hospital Laboratories such as glucose, HbA1c, free tetraiodothyronine (FT4), insulin levels, lipid profile, hs-CRP, and TSH levels. Meanwhile, 2 mL of the residual blood sample was centrifuged (1000 × g, 5 minutes), and the serum was kept at −20°C to examine ANGPTL8 (RR 0.18–3.7 ng/mL) levels and leptin (RR 0.5–15.5 ng/mL) levels. Hormone levels (TSH, insulin and T4) were measured using a fully quantitative ELISA-based chemiluminescent assay (CUSABIO Technology LLC, Houston). All procedures were performed in accordance with the manufacturer’s instructions. Insulin resistance (IR) levels were evaluated by applying the homeostasis model assessment of estimated insulin resistance (HOMA-IR) index = [glucose (mmol/L) × insulin (µU/mL)/22.5], using fasting values of HOMA-IR index < 1.8.^[23]^ The triglyceride glucose (TyG) index was measured by (ln [fasting triglycerides (mg/dL) × fasting glucose (mg/dL)/2]), where standard cutoff values defined for TyG in the literature are approximately 4 to 8.^[24]^

### 2.3. Participants categorization according to thyroid hormones levels

Four types of TD patients were cauterized into: primary hypothyroidism (serum TSH > 4.94 IU/mL and FT4 0.7 ng/dL), primary hyperthyroidism (serum TSH 0.35 IU/mL and FT4 > 1.48 ng/dL), subclinical hypothyroidism (raised TSH with normal FT4 levels), and subclinical hyperthyroidism (decreased TSH). The reference range for TSH was 0.35 to 4.94 IU/mL, while FT4 was 0.7 to 1.48 ng/dL.^[25]^

### 2.4. Anthropometric measurements

BMI was estimated using an electronic scale (Beurer GmbH, Type PS 07, China). The BMI was characterized into below normal (<18.5 kg/m^2^), (b) normal (18.5–24.9 kg/m^2^), and overweight (25.0–29.9 kg/m^2^) or obese (>30.0 kg/m^2^).^[21]^ Waist circumference (WC) was measured using the belly button. Women with WC > 88 cm were considered abdominally obese.^[22]^

Ethical compliance: Ethical approval to perform the study was obtained from the Ethical Committee at the College of Applied Medical Sciences, Taibah University, Madinah, and the Institutional Review Board, General Directorate of Health Affairs in Madinah (IRB 022-22).

Patient consent statement: All participants provided written informed consent for participation in the present study was authorized by the Ethical Committee at the College of Applied Medical Sciences, Taibah University.

### 2.5. Statistical analysis

Graphpad Prism 7 was used for statistics (GraphPad Software, San Diego). Percentages, and mean ± standard deviation (SD) were used to convey quantitative data. Multiple continuous variables were compared using one-way ANOVA. Pearson’s correlation was used to compare ANGPTL8, leptin, thyroid hormone levels, glucose, HbA1c, insulin levels, IR, hs-CRP, BMI, and lipid profile. All differences were statistically considerable at the level of *P* ≤ .05.

## 3. Results

**Figure 1.**
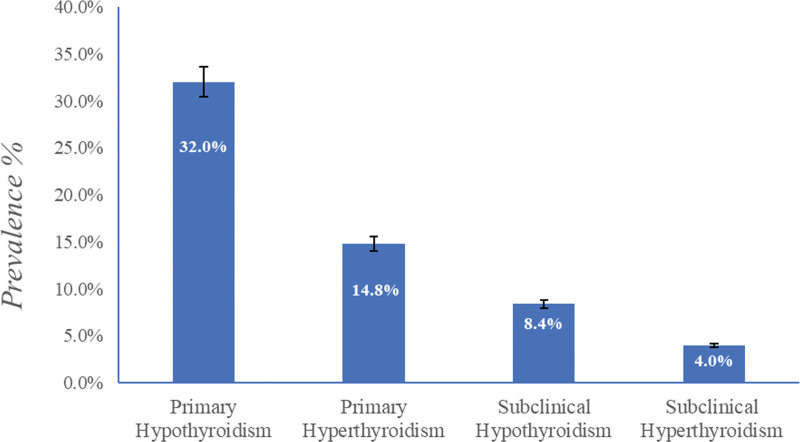
Study population thyroid dysfunction prevalence.

The study indicated the incidence of TD in 59.2% (148) of the study population, with 32% (80) with primary hypothyroidism, 14.8% (37) with primary hyperthyroidism, 8.4% (n = 21) with subclinical hypothyroidism, and 4% (n = 10) with subclinical hyperthyroidism (*P* < .05; Fig. 1).

A total of 250 patients from the Madinah area participated in this study: 32% (80) had hypothyroidism, 14.8% (37) had hyperthyroidism, and 40.8% (102) had no thyroid dysfunction.

All patients were Saudi females with T2DM with an average age of 54.5. The findings suggest that hypothyroidism (7.8 ± 0.67 mmol/L) had higher fasting blood glucose (FBG) levels than hyperthyroidism (7.1 ± 0.64 mmol/L; *P* < .05). High-density lipoprotein cholesterol (HDL-C), triglyceride, TyG index, body mass index (BMI), WC, high-sensitivity C-reactive protein (hs-CRP), and insulin levels exhibited significant differences between females with hypothyroidism and hyperthyroidism (all *P* < .05). Furthermore, the levels of leptin, ANGPTL8, and IR exhibited significant differences (*P* < .05) across the TD groups, as indicated in Table 1.

**Table 1:** Shows general research population characteristics per TD category.

Parameter	All T2DM subjects	Hypothyroidism	Hyperthyroidism	*P* value
	† n = 250(100%)	† n = 80 (32%)	† n = 37 (14.8%)	.05*
Age (yr)	54.5 ± 13.33	55.51 ± 18.11	48.5 ± 12.10	**–**
Duration of diabetes (yr)	11.5 ± 6.6	6.3 ± 3.2	5.2 ± 3.6	**–**
FBG (mmol/L)	7.3 ± 0.77	7.8 ± 0.67	7.1 ± 0.64	**.05** *
HbA1c (%)	7.61 ± 2.46	7.51 ± 2.62	7.55 ± 3.26	>.05
LDL-C (mmol/L)	2.87 ± 0.85	2.85 ± 0.73	2.86 ± 0.97	>.05
HDL-C (mmol/L)	1.02 ± 0.50	0.89 ± 0.52	1.0 ± 0.30	**.03** *
Total cholesterol (mmol/L)	5.88 ± 0.89	5.72 ± 0.87	5.78 ± 0.85	>.05
TG (mmol/L)	2.93 ± 2.84	2.89 ± 2.64	2.79 ± 2.51	**.04** *
(TyG) index	9.7 ± 4.26	9.8 ± 4.22	9.1 ± 4.21	**.05** *
BMI (kg/m^2^)	29.77 ± 8.86	31.5 ± 10.85	25.5 ± 10.84	**.04** *
WC (cm)	120 ± 20.16	125 ± 12.10	110 ± 10.11	**.03** *
Leptin (ng/mL)	26.7 ± 11.11	25.9 ± 10.12	15.5 ± 9.6	**.02** *
ANGPTL8 (ng/mL)	6.99 ± 0.64	5.5 ± 2.12	3.2 ± 1.01	**.03** *
hs-CRP (mg/L)	18.9 ± 5.11	27.9 ± 13.11	20.6 ± 10.13	**.04** *
Fasting insulin (mIU/L)	7.8 ± 6.53	6.8 ± 2.61	8.5 ± 3.54	**.02** *
HOMA-IR index	2.6 ± 1.31	2.3 ± 1.21	2.8 ± 1.41	**.04** *
TSH (µIU/mL)	5.8 ± 2.11	6.2 ± 2.11	0.31 ± 0.15	**.001** **
FT4 (ng/dL)	1.3 ± 0.21	0.5 ± 0.21	1.87 ± 0.81	**.002** **

Data were obtained as the mean ± SD for continuous variables. *P* value was obtained from one-way ANOVA.

Primary hypothyroidism: serum high TSH > 4.94 µIU/mL and low FT4 < 0.7 ng/dL.

Primary hyperthyroidism: serum low TSH < 0.35 µIU/mL and high FT4 > 1.48 ng/dL. Whereas 40.8% (102) of T2DM patients had no thyroid dysfunction.

ANGPTL8 = angiopoietin-like protein 8, BMI = body mass index, FBG = fasting blood glucose, FT4 = free tetraiodothyronine hormone, HbA1C = hemoglobin glycated, HDL-C = high-density lipoprotein cholesterol, HOMA-IR index = insulin resistance index, hs-CRP = high sensitivity-C reactive protein, LDL-C = low-density lipoprotein cholesterol, TG = triglycerides, TSH = thyroid stimulating hormone, TyG index = triglycerides/glucose index, WC = waist circumference.

* *P* ≤ .05.

** *P* ≤ .001.

† Data was presented as numbers (%).

Pearson’s correlation test revealed that there was a substantial positive association between leptin and ANGPTL8 with FBG, TG, TyG index, BMI, hs-CRP, and IR. However, there was a significant negative correlation between leptin and ANGPTL8 with HDL-C levels in both categories of TD.

In hypothyroidism, TSH correlated positively with HbA1c, TG, TyG index, BMI, WC, leptin, ANGPTL8 levels, hs-CRP, and IR but negatively with HDL-C, insulin, and FT4 levels in females with T2DM with hypothyroidism.

FT4 showed negative significant relationships with TG, TyG index, and BMI and a positive link with insulin level in T2DM females with hyperthyroidism. TSH levels were negatively correlated with FBG, HDL-C, hs-CRP, insulin levels, and IR in patients with hyperthyroidism. In females with T2DM and hyperthyroidism, TSH level was significantly positively correlated with TG level, TyG index, and BMI. FT4 correlated positively with insulin levels and IR but negatively with TG, and TyG index in T2DM females with hyperthyroidism (Table 2).

**Table 2:** Pearson’s correlation coefficients between key variables based on thyroid dysfunction (TD) categories.

Hypothyroidism								
Parameter	Leptin		ANGPTL8		TSH		FT4	
	*r*	*P*	*r*	*P*	*r*	*P*	*r*	*P*
FBG (mmol/L)	**0.871**	.001**	**0.671**	.01*	0.231	>.05	0.121	>.05
HbA1c (%)	0.421	>.05	**0.525**	.05*	**0.542**	.05*	0.242	>.05
LDL-C (mmol/L)	0.362	>.05	0.352	>.05	0.242	>.05	0.312	>.05
HDL-C (mmol/L)	**−0.657** †	.04*	0.327	>.05	**−0.512** †	.05*	0.219	>.05
Total cholesterol (mmol/L)	0.417	.06	0.423	.06	0.311	>.05	0.235	>.05
TG (mmol/L)	**0.872**	.002*	**0.572**	.003*	**0.562**	.02*	**−0.537** †	.04*
(TyG) index	**0.632**	.03*	**0.554**	.04*	**0.571**	.04*	**−0.581** †	.03*
BMI (kg/m^2^)	**0.635**	.03*	**0.533**	.05*	**0.554**	.04*	**−0.521** †	.05*
WC (cm)	0.314	>.05	0.311	>.05	**0.516**	.05*	0.221	>.05
Leptin (ng/mL)	1	**–**	**0.543**	.04*	**0.622**	.02*	0.371	>.05
ANGPTL8 (ng/mL)	**0.543**	.04*	1	–	**0.642**	.01*	0.331	>.05
hs-CRP (mg/L)	**0.653**	.03*	**0.562**	.04*	**0.527**	.05*	0.223	>.05
Fasting insulin (mIU/L)	0.122	>.05	0.281	>.05	**−0.632** †	.03*	**0.632**	.02*
HOMA-IR index	**0.872**	.002**	**0.652**	.01*	**0.563**	.02*	0.324	>.05
TSH (µIU/mL)	**0.622**	.02*	**0.642**	.01*	1	–	**−0.843** †	.003**
FT4 (ng/dL)	0.371	>.05	0.331	>.05	**−0.843** †	.003**	1	–
Hyperthyroidism								
FBG (mmol/L)	**0.821**	.001**	**0.631**	.01*	**−0.531** †	.05*	0.131	>.05
HbA1c (%)	0.421	>.05	**0.555**	.05*	0.212	>.05	0.242	>.05
LDL-C (mmol/L)	0.322	>.05	0.252	>.05	0.142	>.05	0.112	>.05
HDL-C (mmol/L)	0.217	>.05	0.127	>.05	**−0.542** †	.05*	0.119	>.05
Total cholesterol (mmol/L)	0.427	>.05	0.433	>.05	0.321	>.05	0.215	>.05
TG (mmol/L)	**0.852**	.004*	**0.562**	.02*	**0.572**	.02*	**−0.547** †	.04*
(TyG) index	**0.631**	.03*	**0.524**	.04*	**0.541**	.04*	**−0.571** †	.03*
BMI (kg/m^2^)	**0.648**	.03*	**0.537**	.05*	**0.534**	.04*	**−0.621** †	.05*
WC (cm)	0.324	>.05	0.315	>.05	**0.519**	.05*	0.251	>.05
Leptin (ng/mL)	1	–	**0.553**	.04*	**0.642**	.02*	0.381	>.05
ANGPTL8 (ng/mL)	**0.553**	.04*	1	–	**0.672**	.01*	0.331	>.05
hs-CRP (mg/L)	**0.655**	.03*	**0.582**	.04*	**–0.557** †	.05*	0.263	>.05
Fasting insulin (mIU/L)	0.192	>.05	0.181	>.05	**−0.652** †	.03*	**0.672**	.02*
HOMA-IR index	**0.875**	.002**	**0.642**	.01*	**−0.593** †	.02*	**0.624**	.04*
TSH (µIU/mL)	**0.642**	.02*	**0.672**	.01*	1	–	**–0.853** †	.003**
FT4 (ng/dL)	0.381	>.05	0.331	>.05	**−0.853** †	.003**	1	–

Bold values indicate positive correlation.

*P* values were obtained from Pearson’s correlation. Starred values point to a significant level.

ANGPTL8 = angiopoietin-like protein 8, BMI = body mass index, FBG = fasting blood glucose, FT4 = free tetraiodothyronine hormone, HbA1C = hemoglobin glycated, HDL-C = high-density lipoprotein cholesterol, HOMA-IR index = insulin resistance index, hs-CRP = high sensitivity-C reactive protein, LDL-C = low-density lipoprotein cholesterol, TG = triglycerides, TSH = thyroid stimulating hormone, TyG index = triglycerides/glucose index, WC = waist circumference.

*P < 0.05.

**P < 0.001.

† Negative correlation.

## 4. Discussion

In Saudi Arabia, the incidences of T2DM and TD are increasing, especially in women.^[1–3,11]^ Therefore, this study was conducted with female living in Madinah, Saudi Arabia, to examine the relationship between TD and T2DM biomarkers (FBG, insulin, ANGPTL8) and its complications, such as obesity (leptin, BMI, WC) and CVD (lipid profile, TyG index, hs-CRP). According to the data presented in Figure 1, it can be observed that 32% of the patients had primary hypothyroidism, whereas 14.8% did not. Similar findings were reported by Hasanato et al^[26]^, who found a significant occurrence of thyroid diseases in the female Saudi adult population, with subclinical hypothyroidism being the most frequent. Alrowaili et al^[27]^ found that females were more likely to have hypothyroidism than men (25.5%). Several studies on T2DM and the incidence of TD have shown that hypothyroidism is more widespread among T2DM patients.^[9–26,28]^ The study findings are consistent with earlier Saudi Arabian and international investigations.^[29,30]^

Furthermore, in patients with hypothyroidism and coexisting T2DM, there was an increase in the levels of TG, leptin, ANGPTL8, and hs-CRP with high BMI, TyG index, and HOMA-IR index. In addition, there was a positive correlation between high TSH levels and high HbA1c, TG, TyG index, BMI, WC, leptin, ANGPTL8levels, hs-CRP, and IR, but a negative correlation with HDL-C, insulin, and FT4 levels in females with T2DM (Tables 1 and 2). Therefore, high TSH and low FT4 levels are associated with a higher risk of T2DM complications. In addition, the results also showed that women with T2DM had elevated level of ANGPTL8 which was significantly correlated with increases in IR and increased levels of C-reactive protein (hs-CRP), TG, and BMI.^[21]^

**Figure 2.**
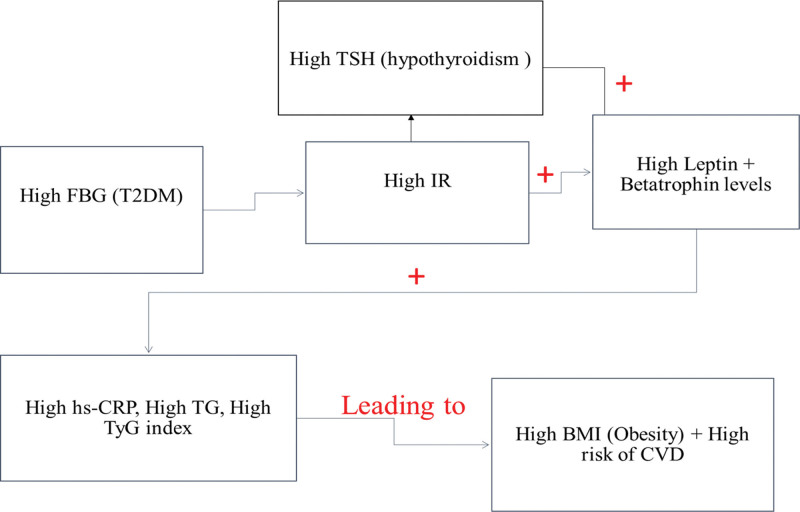
Schematic representation of the association between hypothyroidism and the development of the risk of T2DM complications in a female with coexisting T2DM.

Figure 2 shows the link between high IR and TSH levels and other biomarkers, such as leptin, ANGPTL8 which positively correlated with hs-CRP, TG, and TyG index (a useful predictor for IR and CVD), leading to an increased risk of obesity and CVD. Moreover, researchers reported that in euthyroid and hypothyroidism patients with obesity, elevated TSH was at a higher risk of IR and positively associated with HOMA-IR and atherogenic lipid profiles.^[31]^ In addition, hypothyroidism and diabetes consequences in T2DM have been reviewed,^[32]^ and it was observed that hypothyroidism was more prevalent in T2DM patients than in the general population in a meta-analysis of 36 case-control and cross-sectional studies. Additionally, T2DM with hypothyroidism has an increased risk of developing diabetic nephropathy, retinopathy, and peripheral neuropathy.^[31,33,34]^ Several studies suggest that the diabetes-thyroid link might be bi-directional,^[31,33,34]^ and elevated insulin levels in T2DM may promote thyroid tissue hyperplasia, producing enlargement and nodule development,^[23,34]^ leading to TD, which may impair glucose and insulin metabolism.^[34]^ However, in hyperthyroidism patients with coexisting T2DM, high FT4 levels were found to have a significant positive correlation with high insulin levels and high IR, while a significant negative correlation was detected between high FT4 and TG, TyG index, and BMI in female subjects in current study (Table 2). Hyperthyroidism is characteristically correlated with deteriorating blood glucose and enhanced insulin levels. Excessive thyroid hormones increase glucose production in the liver, quick absorption of glucose through the intestines, and enhance IR.^[6,35–37]^ This information is in line with the results that highlight the strong relationship between high levels of insulin and its resistance with increasing FT4 levels in females with T2DM. In addition, a negative correlation was observed between leptin level, BMI, and high levels of FT4, suggesting that a reduction in weight, as indicated by BMI level, can lead to reduced leptin levels as a result of excessive concentrations of thyroid hormones. A large study observed that leptin levels were decreased in hyperthyroidism compared with a large control group, which was reported as the main outcome for decreasing BMI in hyperthyroidism patients with T2DM.^[38]^ Furthermore, high insulin levels and insulin resistance play important roles in the relationship between hyperthyroidism and T2DM. IR can occur as part of thyroid dysfunction and metabolic abnormalities, and these disorders are recognized as independent risk factors for T2DM and CVD. Also, Type 2 diabetes mellitus (T2DM) is a significant risk factor for the onset of cardiovascular disease (CVD) due to its association with an elevated likelihood of atheroma formation. Additionally, T2DM can impede the development of coronary collateral arteries, which can occur through processes such as angiogenesis and arteriogenesis.^[39]^ In summary, hyperthyroidism and hypothyroidism are 2 examples of TD that may increase the risk of IR and CVD in individuals with T2DM.

### 4.1. Limitations and recommendations

This study had several research limitations. First, as a retrospective study, it might be difficult to find meaningful associations between thyroid dysfunction and T2DM biomarker levels. Prospective studies are needed to determine whether hypothyroidism or hyperthyroidism affects TSH levels and T2DM biomarkers, and their consequences. Second, the sample size of this study was modest; hence, larger prospective investigations are needed.

## 5. Conclusion

The study demonstrated a significant correlation between the presence of type 2 diabetes mellitus (T2DM) in females and a greater prevalence of thyroid dysfunction (TD), particularly hypothyroidism. This link was due to an increase in insulin resistance (IR), which exhibited a positive correlation with thyroid-stimulating hormone (TSH) levels. Moreover, the observed changes in thyroid-stimulating hormone (TSH) levels were correlated with elevated levels of leptin and ANGPTL8, potentially increasing susceptibility to insulin resistance (IR) problems, including obesity and cardiovascular disease (CVD), in individuals diagnosed with type 2 diabetes mellitus (T2DM). Hence, it is imperative to perform routine evaluations for thyroid-stimulating hormone (TSH), free thyroxine (FT4), leptin, and angiopoietin-like protein 8 (ANGPTL8).

Despite these limitations, the current research suggests a possible link between thyroid dysfunction, leptin, and ANGPTL8 levels, and T2DM problems in females with T2DM, advocating regular monitoring of thyroid function, leptin, and ANGPTL8 levels in T2DM patients. The American Association of Clinical Endocrinologists (AACE) and the American Thyroid Association (ATA) have advised routine monitoring of thyroid function in T2DM patients.^[16]^ Moreover, consistent with the guidelines of the European Thyroid Association, monitoring of thyroid function should be performed manually in T2DM patients.

Furthermore, the prompt initiation of thyroid therapy is recommended in the first phase. To mitigate or minimize detrimental cardiovascular outcomes, it is imperative to monitor glucose and insulin levels consistently.

## Acknowledgments

The authors extend their appreciation to the Deputyship for Research & Innovation, Ministry of Education in Saudi Arabia for funding this research work through the project number WE-44-0103. The authors would also be grateful to Miss Johayna Aboalkayer (Taibah University, Medical Applied Science College’s technician), the lab technician who supported and assisted us in the present study.

## Author contributions

**Conceptualization:** Walaa Mohammedsaeed.

**Data curation:** Dalal Binjawhar, Walaa Mohammedsaeed.

**Formal analysis:** Walaa Mohammedsaeed.

**Funding acquisition:** Dalal Binjawhar.

**Investigation:** Dalal Binjawhar, Walaa Mohammedsaeed.

**Methodology:** Walaa Mohammedsaeed.

**Validation:** Walaa Mohammedsaeed.

**Writing – original draft:** Walaa Mohammedsaeed.

**Writing – review & editing:** Dalal Binjawhar, Walaa Mohammedsaeed.
